# F21. ELECTROPHYSIOLOGICAL PARAMETERS OF SELECTIVE ATTENTION IN ADOLESCENTS WITH A FIRST EPISODE OF PSYCHOSIS: A COMPARISON WITH ADHD

**DOI:** 10.1093/schbul/sby017.552

**Published:** 2018-04-01

**Authors:** Iris Selten, Jacob Rydkjaer, Anne Katrine Pagsberg, Birgitte Fagerlund, Birte Glenthoj, Jens Richardt Møllegaard Jepsen, Bob Oranje

**Affiliations:** 1 CINS & CNSR, Mental Health Centre Glostrup, University of Copenhagen; 2 CINS & CNSR, Psychiatric Center Glostrup; 3 Child and Adolescent Mental Health Services; 4 Center for Neuropsychiatric Schizophrenia Research (CNSR) and Center for Clinical Intervention and Neuropsychiatric Schizophrenia Research (CINS), Copenhagen University Hospital; 6 CINS and CNSR, University of Copenhagen/University Medical Center Utrecht

## Abstract

**Background:**

Neuropsychological deficiencies in attentional processes and filtering of information are shown by both patients with schizophrenia and Attention Deficit Hyperactivity Disorder (ADHD). Given that behavioral symptoms differ, differential neurophysiological processes are likely to be underlying each disorder. Deficiencies in early auditory processing measured by event-related potentials (ERPs) such as the P300 amplitude and mismatch negativity are suggested to be biomarkers for schizophrenia. Here we study if these electrophysiological processes are impaired in, and specific for, young individuals with a first episode psychosis (FEP), by directly comparing them with typically developing peers and adolescents with ADHD.

**Methods:**

27 FEP patients, 25 ADHD patients and 45 age and gender matched Healthy Controls (HC), all aged between 12 and 17 years old, were assessed for their N1, N2, P2, P3a, P3b, MMN and PN amplitudes in a selective attention paradigm (auditory oddball task).

**Results:**

ADHD patients showed significantly smaller N2 and P3b amplitudes than HC, whereas FEP patients showed intermediate amplitudes. In addition, we found a second order interaction effect, indicating that the HC group had larger P2 amplitudes to attended deviant stimuli than to unattended deviant stimuli, whereas the response to non-attended deviant stimuli did not differ from that to non-attended standard stimuli. The ADHD group did not show this difference, in fact, they showed P2 amplitudes to unattended deviant stimuli that were larger than those to unattended standards. Interestingly, this effect was also found in the FEP group, although this only reached trend level (p = 0.08) of statistical significance. Post-hoc dividing the FEP group in patients with and without a diagnosis of schizophrenia, showed the same second order interaction effect in patients without schizophrenia as that in the ADHD subjects, while the interaction effect in patients with schizophrenia was identical to that of the HC. Furthermore, FEP patients without schizophrenia showed significantly smaller P3b amplitudes compared to FEP patients with schizophrenia and HC. Last, FEP patients with schizophrenia showed trend level significant smaller MMN amplitudes than HC (p = 0.09). No further significant group differences were found.

**Discussion:**

FEP patients without schizophrenia showed impaired neurophysiological functioning compared to their counterparts with schizophrenia, who in turn showed similar performance as healthy controls. Given that use of medication did not differ between FEP patients with and without schizophrenia, our data suggest that other factors may play a role, such as comorbid symptoms. Since we found considerable overlap between FEP patients without schizophrenia and ADHD patients our current data do not support the theory that any of the investigated electrophysiological measures are useful as specific biomarkers for schizophrenia.

